# Medication and fall injury in the elderly population; do individual demographics, health status and lifestyle matter?

**DOI:** 10.1186/1471-2318-14-92

**Published:** 2014-08-23

**Authors:** Björg Helgadóttir, Lucie Laflamme, Joel Monárrez-Espino, Jette Möller

**Affiliations:** 1 Department of Public Health Sciences, Karolinska Institutet, Stockholm, Sweden

**Keywords:** Fall injury, Medications, Lifestyle, FRIDs, Cohort study, Older people

## Abstract

**Background:**

The simultaneous use of several medications is an important risk factor for injurious falls in older people. The aim of this study is to investigate the effect of the number of medications dispensed to elderly persons on fall injuries and to assess whether this relationship is explained by individual demographics, health habits and health status.

**Methods:**

A population-based, nested, case–control study on people 65 years and older (N = 20.906) was conducted using data from the Stockholm Public Health Cohort (SPHC) derived from self-administered surveys and linked at the individual level with various Swedish health registers. Fall injuries leading to hospitalization recorded in the Swedish National Patient Register (NPR) were considered as the outcome. The main exposure, obtained from the Swedish Prescribed Drug Register (SPDR), was the number of medications dispensed within 90 days prior to the injurious fall. The injury risk was estimated using adjusted odds ratios (ORs) from logistic regression. Results were adjusted by selected demographic, social circumstances, lifestyle and health status data extracted from the SPHC.

**Results:**

After adjusting for common risk factors within demographics, lifestyle, social circumstances and health status, using more than one medication increased the risk of fall injury but no clear dose–response relationship was observed, with point estimates ranging from 1.5-1.7 for the use of two, three, four or five or more medications as compared to using none. An increased risk remained, and was even elevated, after adjusting for the use of fall-risk-increasing drugs (FRIDs).

**Conclusions:**

Using more than one medication affects the risk of injurious falls among older people. The effect of any given number of medications studied remains and is even strengthened after adjusting for individual demographics, health habits, health conditions and the use of FRIDs.

## Background

Between 30 and 60% of the elderly community-dwelling population in high-income countries fall every year [1]. Injurious falls cause considerable morbidity and mortality [2], and can have wide-ranging negative individual and societal consequences [1,3]. Medications are an acknowledged risk factor for falls; not only do some types of medication play a role [4-6], e.g. fall-risk-increasing drugs (FRIDs) [7], but even combinations – or number – of medications [8-10], in particular polypharmacy [11,12], also have an effect. Whether the effect of the number of medications is merely due to the use of FRIDs [7] or rather a reflection of other mechanisms remains to be established.

The association between number of medications and injurious falls is complex and can be influenced by a range of other individual risk factors. Among those are individual demographic attributes such as gender and age, and health status [1,13-15]. Healthy lifestyles are regarded as protective against falls and fall-related injuries among older people [16-18]. Physical activity, for its part, increases muscle strength and improves balance, thereby reducing the risk of both falls and fractures [19]. The association between alcohol consumption and injuries among older people [20], for its part, may be both direct or due to potential interactions with some medications [21,22]. Studies reveal that poorer socioeconomic status is associated not only with poorer health but also with higher rates of injuries [13,15,23]. Although some studies suggest that the strength of the association is lower among the elderly and can indeed be reversed [23], the evidence at hand remains scarce and largely descriptive.

Previous studies investigating FRIDs and polypharmacy have had small sample sizes, ranging from N = 118 to 293 [8,9,11,12], with two exceptions where the samples were larger (N = 6928 and N = 11640) [7,10], prompting the need for larger studies with more opportunities to adjust for a broader range of confounders. Additionally, studies using fall injuries rather than just falls as an outcome are not so common, despite the fact that fall injuries could conceivably be considered as the more interesting outcome, due to the larger risk of future health consequences. This study investigates the effect of the number of medications on fall injuries among older people and assesses to what extent this relationship is affected by individual demographics, health habits and health status.

## Methods

We designed a population-based, case–control study nested within a cohort using data from the Stockholm Public Health Cohort (SPHC) [24]. The SPHC includes three cohorts recruited through the Public Health Surveys (PHS) conducted in 2002, 2006 and 2010 respectively, and data-linked at the individual level to the Swedish Total Population Register, the National Patient Register (NPR) and the Swedish Prescribed Drug Register (SPDR) using the Swedish personal identity number, a unique identification number assigned to all residents of Sweden. The PHSs consist of area-stratified random samples of the Stockholm County population aged 18–84 years (in 2002 and 2006) and ≥18 years (in 2010). For this study, we selected the respondents who answered the PHS in 2010 and were 65 years and older (N = 21 747, response rate 73.5%) [24].

### Cases and controls

Cases were defined as those that were hospitalized for injuries as a result of a fall during the 16-month period from 1 September 2010 to 31 December 2011 (N = 498). This information was retrieved from the NPR using discharge diagnoses classified according to the ICD-10 codes W00-W19. Date of admission was set as the index date. The most common reasons for hospitalization were injuries to the hip or thigh area (32.9%), followed by head injuries (11.2%), injuries to the shoulder or upper arm (9.4%), injuries to the abdomen, lower back, lumbar spine and pelvis (8.4%), injuries to the knee or lower leg (7.4%), injuries to the thorax (5.4%), and injuries to the elbow or forearm (5.0%).

Controls were defined as all those who did not have an injurious fall, according to information from the NPR, during the study period (N = 21 249). Controls were assigned a random index date within the follow-up period. For some controls (n = 227 (1.1%)), this date fell on a date after a control had died and therefore was replaced with the date they answered the PHS in 2010.

Individuals (both cases and controls) with injurious falls leading to hospitalization in the 12-month period prior to the survey date were excluded, in order to avoid reversed causation. The final sample included 464 cases and 21 004 controls.

### Medications

Information on medication use was extracted from the SPDR where all dispensed prescriptions in ambulatory care (regardless of whether the drug is subsidised or not) have been registered for all residents in Sweden since July 2005. The register contains information on the medication name, dosage and dates for when the drug was prescribed and dispensed [25]. All dispensations within the 90-day period prior to the index date were accounted for. The number of different dispensed medications was calculated based on the full five-level Anatomical Therapeutic Chemical (ATC) code. The ATC classification system was devised by the World Health Organization and it categorizes medications based on which system or organ they act upon in the body and their therapeutic and chemical features [26].

Fall-risk-increasing drugs (FRIDs) were considered as a potential confounder in the relationship between number of medications and fall injury. FRIDs were defined following the classification used by the Swedish National Board of Health and Welfare [27]. The list of FRIDs with their corresponding ATC codes is as follows: vasodilators used in cardiac diseases (C01D), antihypertensive drugs (C02), diuretics (C03), beta blocking agents (C07), calcium channel blockers (C08), agents acting on the renin-angiotensin system (C09), alpha-adrenoreceptor antagonists (for benign prostatic hypertrophy) (G04CA), and opioids (N02A) dopaminergic agents (anti-Parkinson drugs, N04B), antipsychotics excl. lithium (N05A excl. N05AN), anxiolytics (N05B), hypnotics and sedatives (N05C) and antidepressants (N06A). The calculated number of FRIDs during the 90-day period prior to the index date was categorized into two groups: 0 vs. ≥1.

### Covariates

The covariates considered fall into the following five domains: demographics, housing and household finance, lifestyle, social circumstances and health status. These domains were inspired by a report on the health and life-situation of elderly people in Stockholm [28]. The variables included in each domain are clarified below and can also be found in the first column of Table 1. All of the covariates were measured in the 2010 PHS or in the Swedish Total Population Register at baseline.

**Table 1:** Characteristics of the participants and the association with risk of fall injury

	**Controls**		**Cases**			
**Exposures**	**%**	**(n)**	**%**	**(n)**	**Crude OR (95% CI)**	**OR adjusted for age and sex (95% CI)**
***Demographics***						
Age (in years)						
*65-69*	37.0	(7597)	13.0	(60)	1.00	*(REF)*
*70-74*	25.0	(5140)	12.5	(58)	1.20 (0.79-1.81)	*1.19 (0.79-1.81)*
*75-79*	16.9	(3474)	17.5	(81)	**2.76** (1.87-4.07)	***2.72*** *(1.85-4.01)*
*80-84*	12.2	(2494)	20.1	(93)	**4.86** (3.34-7.08)	***4.77*** *(3.27-6.94)*
*≥85*	8.9	(1820)	36.9	(171)	**10.79** (7.64-15.24)	***10.49*** *(7.43-14.79)*
Gender						
*Male*	47.2	(9680)	35.6	(165)	1.00	*(REF)*
*Female*	52.8	(10845)	64.4	(298)	**1.58** (1.27-1.95)	***1.41*** *(1.14-1.75)*
Country of origin						
*Sweden*	84.8	(17395)	85.1	(394)	1.00	(REF)
*Other Nordic countries*	7.4	(1528)	7.8	(36	1.03 (0.70-1.52)	1.14 (0.77-1.69)
*Other European countries*	5.7	(1168)	5.4	(25)	0.93 (0.59-1.46)	0.92 (0.57-1.43)
*The rest of the world*	2.1	(434)	1.73	(8)	0.86 (0.41-1.80)	1.05 (0.49-2.23)
Marital status						
*Married*	57.3	(11767)	39.3	(182)	1.00	*(REF)*
*Unmarried*	7.2	(1474)	7.1	(33)	1.47 (0.98-2.20)	***1.56*** *(1.04-2.35)*
*Divorced*	18.1	(3707)	16.0	(74)	1.21 (0.88-1.65)	*1.24 (0.90-1.70)*
*Widowed*	17.4	(3577)	37.6	(174)	**3.05** (2.41-3.86)	***1.38*** *(1.05-1.80)*
***Housing and finance***						
Type of housing						
*Own house*	40.2	(8126)	26.2	(117)	1.00	*(REF)*
*Own apartment*	34.4	(6962)	35.2	(157)	**1.50** (1.14-1.97)	*1.10 (0.84-1.45)*
*Rental accommodation*	23.0	(4643)	32.5	(145)	**1.99** (1.51-2.62)	*1.30 (0.98-1.72)*
*Assisted living*	1.3	(263)	5.4	(24)	**6.20** (3.76-10.21)	***2.01*** *(1.18-3.41)*
*Other*	1.2	(240)	0.7	(3)	0.94 (0.29-3.02)	*0.52 (0.16-1.69)*
Presence of^1^:						
Financial stress - general	5.5	(1061)	8.6	(36)	1.44 (0.97-2.12)	**1.62** (1.09-2.42)
Financial stress – health care	5.2	(1002)	5.8	(24)	1.06 (0.66-1.71)	1.37 (0.84-2.23)
***Lifestyle***						
Presence of^1^:						
<3 hours of exercise per week	73.3	(14469)	84.1	(354)	**1.97** (1.46-2.65)	**1.52** (1.12-2.07)
<2 hours of housework per day	60.4	(12086)	71.9	(317)	**1.53** (1.21-1.93)	**1.56** (1.22-1.99)
< 20 minutes of walking/cycling per day	26.6	(5333)	43.9	(194)	**2.19** (1.77-2.71)	**1.69** (1.35-2.10)
Sedentary behaviour	12.5	(2451)	28.7	(121)	**2.76** (2.17-3.50)	***2.05*** *(1.59-2.65)*
Alcohol binge drinking	7.1	(1413)	9.1	(40)	1.18 (0.82-1.70)	***1.84*** *(1.26-2.69)*
Daily smoking	9.7	(1921)	11.8	(52)	1.21 (0.87-1.69)	***1.66*** *(1.18-2.32)*
Daily smokeless tobacco use	4.1	(796)	2.8	(12)	0.64 (0.33-1.24)	1.12 (0.56-2.22)
***Social circumstances***						
Presence of^1^:						
Living alone	33.9	(6836)	57.0	(254)	**2.37** (1.92-2.93)	***1.45*** *(1.14-1.84)*
Distrusting the neighbours	4.5	(903)	6.5	(29)	1.44 (0.94-2.21)	**1.57** (1.01-2.44)
Lack of social support	12.8	(2580)	14.4	(64)	1.13 (0.84-1.51)	1.20 (0.89-1.61)
Low participation in social activities	41.4	(8246)	59.4	(260)	**2.12** (1.71-2.63)	***1.72*** *(1.38-2.16)*
***Health status***						
BMI						
*Underweight*	1.8	(350)	4.9	(20)	**2.31** (1.37-3.90)	*1.52 (0.88-2.61)*
*Normal weight*	45.5	(8868)	56.0	(229)	1.00	*(REF)*
*Overweight*	39.7	(7725)	31.5	(129)	**0.65** (0.51-0.83)	*0.80 (0.63-1.02)*
*Obesity*	13.1	(2542)	7.6	(31)	**0.42** (0.28-0.65)	***0.56*** *(0.36-0.85)*
Presence of^1^:						
Poor self-rated health	6.3	(1250)	19.4	(85)	**3.64** (2.78-4.78)	**2.56** (1.91-3.43)
Long-term limiting disease	38.4	(7515)	57.8	(241)	**2.35** (1.89-2.93)	**1.83** (1.45-2.30)
Persistent fatigue	30.3	(5794)	45.3	(182)	**2.03** (1.63-2.54)	**1.50** (1.19-1.91)
Sleep problems	39.0	(7568)	51.5	(213)	**1.63** (1.31-2.03)	**1.43** (1.14-1.80)
Reduced mobility	27.3	(5466)	59.4	(265)	**3.87** (3.12-4.79)	***2.34*** *(1.85-2.95)*
Pain or discomfort	60.0	(11913)	76.0	(335)	**2.13** (1.67-2.72)	**1.67** (1.30-2.15)
Poor mental health (GHQ-12 ≥ 3)	11.0	(2216)	25.9	(114)	**2.74** (2.15-3.48)	***2.12*** *(1.64-2.74)*
Depression diagnosis	7.7	(1491)	9.0	(38)	1.19 (0.82-1.73)	1.33 (0.91-1.93)
Diabetes diagnosis	11.8	(2386)	13.1	(59)	1.10 (0.81-1.50)	1.18 (0.87-1.61)
Chronic obstructive pulmonary disease diagnosis	5.3	(1070)	9.1	(40)	**1.62** (1.13-2.33)	1.38 (0.95-2.01)
Angina diagnosis	9.8	(1938)	15.8	(68)	**1.66** (1.24-2.23)	1.24 (0.92-1.68)
Congestive heart failure diagnosis	7.8	(1547)	17.8	(77)	**2.39** (1.81-3.16)	**1.50** (1.12-2.01)
High blood pressure	49.2	(9958)	53.6	(241)	1.20 (0.97-1.48)	1.01 (0.82-1.26)

**Bold** represent significant ORs at the p < .05 level while ORs in ***bold italics*** represent risk factors that were entered into the final model after testing as described in the methods.

^1^The absence of the characteristic was used as a reference group.

### Demographics (four variables)

1) *age-group (in five categories)*; 2) *sex*; 3) *country of birth; 4) marital status*.

### Housing and finances (three variables, all but the first variable dichotomized)

1) *type of housing*; 2) *financial stress - general*, i.e. having had problems in the last 12 months paying for housing, food, bills etc.; 3) *financial stress - health care*, i.e. having refrained from going to the dentist, seeking health care or taking out prescriptions in the past 12 months because of financial difficulties.

### Lifestyle (seven dichotomized variables)

1) *<3 hours of exercise per week*; 2) *<2 hours of housework per day*; 3) *< 20 minutes of walking/cycling per day*; 4) *sedentary lifestyle*, i.e. sitting the majority of the time during daily activities; 5) *alcohol binge drinking*, i.e. drinking at least one bottle of wine (or corresponding amount) at least once a week in the last 12 months; 6) *daily smoking*; 7) *daily smokeless tobacco use*.

### Social circumstances (four dichotomized variables)

1) *living alone*; 2) *distrusting the neighbours*; 3) *low social support*, i.e. feeling as if you do not get support from others in times of trouble or crisis; 4) *low participation in social activities*, i.e. does not participate regularly in activities with other people.

### Health status (14 variables, all but the first variable dichotomized)

1) *body mass index (BMI)* based on self-reported height and weight, underweight BMI < 18.5, normal weight BMI between 18.5-24.9, overweight BMI between 25–29.5, and obesity BMI ≥30; 2) *poor self-rated health*, i.e. responding that they have poor or very poor health when asked: “how would you regard your general health status?”; 3) *long-term limiting disease*, i.e. having a long-term disease, health problem or handicap that limits daily activities; 4) *persistent fatigue*, i.e. the presence of persistent fatigue that causes mild or severe discomfort; 5) s*leep problems*, i.e. the presence of sleep problems that causes mild or severe discomfort; 6) *reduced mobility*, i.e. either walking with difficulty or being bedridden; 7) *pain or discomfort*, i.e. the presence of moderate or severe pain or discomfort; 8) *poor mental health*, i.e. scoring ≥3 on the General Health Questionnaire (GHQ-12) which is a validated scale for measuring mental health [29]; 9) *depression diagnosis*; 10) *diabetes diagnosis* (type not specified)*;* 11) *chronic obstructive pulmonary disease diagnosis*; 12) *angina diagnosis*; 13) *congestive heart failure diagnosis*; 14) *high blood pressure*, i.e. being treated for high blood pressure at the time of the questionnaire. All of the diagnosis variables (9–13) were based on the question: “Have you, by a physician, been given any of the following diagnoses…?” We included the health status variables that were available in the PHS and also likely to be confounders based on previous studies.

### Statistical analyses

Data was reweighted to minimize the impact of non-response using calibration weights, provided by the SPHC research group. These weights are based on available variables from various Swedish registers, and include information on sex, age, country of birth, marital status, income, educational level, sickness allowance and area of residence [24]. The data was analysed using multivariate logistic regression, taking calibration weights for non-response and the stratification sampling method into consideration. All results presented in tables are based on those individuals that had information on weights and stratification (4 cases and 558 controls were excluded).

The association between number of medications and risk of fall injury was investigated using multivariate logistic regression as described above; adjusted for sex and age, and the use of FRIDs.

To prevent co-linearity, we performed a correlation analysis between the variables within each domain using Chi^2^-tests and Cramer’s V coefficients. The variables that scored higher than 0.3 on Cramer’s V were investigated further, hence we selected the uncorrelated variables that had the largest effect on the relationship between number of medications and risk of fall injury, which led to the exclusion of financial stress - health care, poor self-rated health, long-term limiting disease and persistent fatigue. The covariates were dealt with in three steps to determine which should be included in the final model. The first step was to test the association between fall injury and each covariate separately, adjusting for sex and age using logistic regression. The covariates that showed a significant effect (p < .05) were tested further in the second step by adding them into a model for the association between number of medications and risk of fall injury. In the third step, the remaining significant covariates were added into the model together with the other significant covariates in the same risk factor domain. Those covariates that were still significant after the third step were included in the final model. The final model was then further adjusted by FRIDs (0 vs. ≥1) to investigate if there was an effect remaining after adjusting for FRIDs. We also investigated whether seasonality had an effect on risk of falling, after adjusting for sex and age, as people might be more likely to fall at certain times of the year due to weather conditions (results not shown). No such association was found and hence seasonality was not further adjusted for. Statistical significance for all analysis was set at α = 0.05.

All statistical analyses were performed in SAS software version 9.3 for Windows (SAS institute Inc., Cary, NC, USA).

The study was approved by the Stockholm Ethical Review Board (2011/344-31/5).

## Results

**Figure 1:**
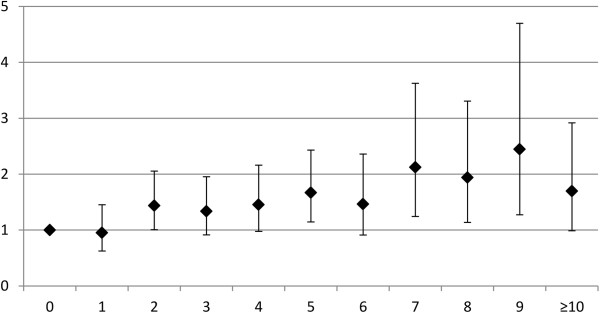
The effects of medications on the risk of fall injuries adjusted by sex and age (OR’s with 95% CIs).

Figure 1 represents the association between number of medications and fall injury adjusted by age and sex. It illustrates that the point estimates for risk of fall injury tend to increase the greater the number of medications, but the confidence intervals overlapped, suggesting that there is no statistically significant trend.

The last two columns in Table 1 represent the crude and adjusted odds ratios for the association between each covariate and risk of fall injury. People over 70 years had an increased risk even after adjustment for sex. The majority of injured fallers were women (64%) who had a higher risk of falling compared to men (OR 1.4, 95% CI 1.1, 1.8). There was also an increased risk for general financial stress (OR 1.6, 95% CI 1.1, 2.4) but not for financial stress due to health care. All of the lifestyle factors, e.g. being sedentary (OR 2.1, 95% CI 1.6, 2.7) or engaging in binge drinking (OR 1.8, 95% CI 1.3, 2.7), were associated with fall injury except using smokeless tobacco. Being socially isolated was also associated with elevated risk estimates, e.g. living alone (OR 1.5, 95% CI 1.1, 1.8) or distrusting the neighbours (OR 1.6, 95% CI 1.0, 2.4). Obesity was protective against fall injury (OR 0.56, 95% CI 0.36, 0.85), but other health status factors such as poor self-rated health (OR 2.6, 95% CI 1.9, 3.4) or reduced mobility (OR 2.3, 95% CI 1.9, 3.0) had elevated risk estimates. Although poor mental health doubled the risk of fall injury (OR 2.1, 95% CI 1.6, 2.7), having been diagnosed with depression did not. In fact, the only diagnosis that showed an increased risk was congestive heart failure (OR 1.5, 95% CI 1.1, 2.0). Others, such as angina, COPD, diabetes and high blood pressure did not.

The variables with italicized ORs in the final column (Table 1) represent those risk factors that remained significant in the model for the association between number of medications and fall injury after testing (see methods) and were included in the final models. These were: marital status, type of housing, sedentary behaviour, alcohol binge drinking, daily smoking, living alone, low participation in social activities, BMI, reduced mobility and poor mental health.

The accumulated number of medications increased the risk of fall injury, though this association was partly explained by age and sex (Table 2). After adjusting for the covariates, it became apparent that there seems to be a threshold as the point estimates are similar for using two, three, four and five or more medications. After further adjustment for FRIDs, the elevated risk estimates for number of medications remained and was even strengthened, with no clear dose–response relationship seen.

**Table 2:** The association between number of medications and the risk of fall injury

	**Controls**		**Cases**					
**Medications**	**%**	**(n)**	**%**	**(n)**	**Crude OR (95% CI)**	**ORs (95% CI) adjusted for age and sex**	ORs (95% CI) adjusted for age, sex and other risk factors*	ORs (95%CI) adjusted for age, sex, other risk factors* and the use of FRIDs
All medications								
*0 medication*	40.7	(8318)	28.3	(130)	1.00	(REF)	1.00 (Ref)	1.00 (Ref)
*1 medication*	12.0	(2443)	7.4	(34)	0.91 (0.60-1.38)	0.95 (0.63-1.45)	1.26 (0.75-2.11)	1.40 (0.82-2.40)
*2 medications*	11.2	(2283)	11.7	(54)	**1.63** (1.14-2.32)	**1.44** (1.01-2.05)	**1.74** (1.10-2.77)	**2.06** (1.16-3.64)
*3 medications*	9.8	(1998)	9.6	(44)	**1.58** (1.09-2.31)	1.34 (0.91-1.95)	1.50 (0.94-2.41)	1.83 (0.94-3.57)
*4 medications*	8.0	(1636)	9.8	(45)	**1.82** (1.24-2.68)	1.45 (0.98-2.16)	**1.74** (1.07-2.81)	**2.14** (1.13-4.05)
*≥5 medications*	18.4	(3768)	33.3	(153)	**2.58** (1.98-3.36)	**1.79** (1.36-2.35)	**1.53** (1.06-2.21)	**1.92** (1.08-3.41)

*Marital status, type of housing, sedentary behaviour, alcohol binge drinking, daily smoking, living alone, low participation in social activities, BMI, reduced mobility and poor mental health.

**Bold** represent significant ORs at the p < .05 level.

## Discussion

To date the association between number of medication and injurious falls has mainly dealt with polypharmacy, usually defined as five medications or more [8,10-12,30] and found that it is a risk factor in itself. Our study shows that, compared to not using any prescribed medication, the use of any number above one leads to almost a two-fold increased risk of falls leading to hospitalization, even after controlling for a range of potential confounders and taking into account that some of the medications can be FRIDs. We do not find however any dose response relationship. This contrasts to an earlier meta-analysis showing that each additional medication increased the risk of falls [13], but where control for confounding was limited. Early studies assessing the effect of polypharmacy on falls among older people reveal mixed results, with chronic disease and multiple pathology fully explaining the association with falls [31], and poor health status not remarkably affecting the association among geriatric outpatients [12]. In this study, the group of diseases considered, including depression, high blood pressure and COPD, did not affect the association observed. When adjusting for those individual confounders that remained significant – socio-demographic (marital status, type of housing), behavioural (e.g., sedentary behaviour, alcohol binge drinking, daily smoking), and health-related (e.g., BMI, poor mental health) – a significant association remained. The finding that obesity may lower the risk of injurious falls contrasts with results indicating that increased BMI can be associated with poor balance [32] and thereby lead to falls. But whether this in turn leads to an increased risk of injurious falls, serious enough to lead to hospitalization, as is the case in our study, remains to be determined. Indeed, studies indicate that obesity can protect against severe injuries [33].

An additional finding of interest is that, after considering the use of FRIDs, the association found not only remained but was also strengthened. This contrasts with earlier findings among people aged 55 years and older where polypharmacy only had an effect if at least one of the drugs used was a FRID [7] or among nursing home residents where polypharmacy was only a risk factor when combined with using a FRID [11]. Whether and to what extent the difference between studies is a result of the difference in study populations or in the list of FRIDs considered remains to be determined. It appears however that the association between number of medications and fall injuries in older people can be manifest in different target groups from small numbers of medications and that the mechanisms lying behind the association are rather complex. The reason for finding that even the use of two medications or more increases the risk of fall injury might be that a number of FRIDs are yet to be identified. Alternatively, this might be a reflection of an interaction between a medication and vulnerability for falls due to disease, which might not have been well captured by the data at hand. On the other hand it could be that the use of prescribed medications, that on their own are regarded as safe, but in combination could produce side-effects that have not been explored. In our study, we did not have access to over-the-counter medications that could also potentially produce precarious side-effects when combined with prescribed medications.

This study used a large, population-based sample and allowed for the consideration of a range of covariates. Using a case–control design nested in cohort data minimized the recall bias. Outcome and exposure data were retrieved from registers. For the former, the inpatient register has almost complete coverage, with accurate data on injury date. We also have accurate data on number and type of prescribed medications at the time of the fall equally for all SPHC participants. Fall injury leading to hospitalization as an outcome is more homogeneous than fall in general – and less subject to recall bias – but indeed less common. This explains the lower prevalence in our study (2.2%), in comparison with rates reported at population level; around one out of three falls among older people requires treatment [34], and a fraction require hospitalization.

We did minimize the impact of non-response bias by adjustments using weights, but some bias may remain. We believe the results apply to relatively healthy older community dwellers in Sweden but the extent to which they apply to the most ailing people is uncertain, including those who are frail and/or nursing home residents [11,35]. It’s worth noting that Sweden has a very low rate of institutionalization among the elderly [36].

Despite the very rich and extensive material at hand, some residual confounding might exist. Additional potential confounders include poor eyesight, more detailed and objective measures of mobility, diseases not captured by the survey (e.g., severe central nervous system diseases). Further, we did not adjust for length of stay to avoid collinearity with number of medications.

Our definition of FRIDs was based on the most recent guidelines from the Swedish National Board of Health and Welfare, which are derived from expert opinion and literature reviews. It is uncertain whether a broader – or different – definition of FRIDs would affect our findings. Also of interest in this respect is that the list of FRIDs considered herein includes analgesics.

Finally, we employed a 90-day interval to capture recent medication as the normal interval for drug prescription in Sweden is three months. By doing so, medication prescribed on any other basis (i.e. when needed or for longer time intervals) may not have been captured and the total number of medications underestimated. The same applies to eventual use of over-the-counter medicines (OTC) in elderly people [37] but there is no obvious reason why they would be differentially used between cases and controls.

## Conclusions

This study revealed that not only polypharmacy but also a small number of medications can have adverse effects on older people’s safety, which may overshadow their desired positive effects. That the use of two or more medications, independently of FRID or not, is a risk factor for injurious falls is a serious concern as falls are prevalent and often have severe consequences for older people. These kinds of associations need to be acknowledged so that physicians and patients can make informed decisions when prescribing and using medications. From a public health perspective, our results fuel the ongoing discussions on requirements and existing guidelines concerning the review of medications being prescribed to older people, not least those still living in the community. Needless to say, this is quite imperative as medications are seldom tested on elderly populations and pharmacokinetics and pharmacodynamics processes may be overlooked [38].

## Competing interests

The authors declare that they have no competing interests.

## Authors’ contributions

All authors contributed to the definition of the research questions, the design of the study and the interpretation of the results. BH conducted the statistical analyses and wrote the first draft of the manuscript, which LL, JME and JM helped to finalize. All authors read and approved the final manuscript.

## Pre-publication history

The pre-publication history for this paper can be accessed here:

http://www.biomedcentral.com/1471-2318/14/92/prepub
